# Psychological, social and pharmacological prevention and interventions for young people with emerging, subthreshold or diagnosed personality disorders: A systematic review

**DOI:** 10.1007/s00787-026-03030-6

**Published:** 2026-04-28

**Authors:** Rona Murad, Kiana Pollard, Naomi Glover, Vivien Wong, Sarah Rowe

**Affiliations:** 1 Division of Psychiatry, University College London, London, United Kingdom; 2 Athar Medical Centre, Manama, Kingdom of Bahrain; 3 Department of Clinical, Educational and Health Psychology, University College London, London, United Kingdom

**Keywords:** Child, Adolescent, Emerging personality disorders, Intervention, Prevention, Personality disorders

## Abstract

Despite increasing recognition of personality disorder traits in adolescence, there is currently no systematic review that synthesises evidence across all intervention types and across the full spectrum of personality disorders in young people. This systematic review aims to describe existing psychological, psychosocial and pharmacological prevention and interventions for young people with PDs. Medline, Embase, PsycINFO, and Web of Science were used to retrieve studies. Effective Public Health Practice Project and Critical Skills Appraisal tools was used to assess risk of bias. Studies were included if they examined a prevention or intervention for individuals up to and including the age of 25 years with a PD. Findings were analysed using a narrative synthesis. Overall, forty-three studies were included. Thirteen studies evaluated Dialectical Behaviour Therapy, mostly for borderline PD (BPD), with six reporting significant symptom reductions (*p* < 0.01). Findings for self-harm and suicidal ideation were mixed. Mentalisation Based Therapy was examined in nine studies, with some evidence of reduced BPD symptoms and self-harm. Interventions such as Metacognitive Interpersonal Therapy (Group)-, Cognitive Analytic Therapy, and Emotion Regulation Training showed promising but inconsistent results. Two randomised controlled trials of omega-3 fatty acids and one of intranasal oxytocin suggested potential benefits, though evidence remains limited. This review suggests that future research should focus on investigating preventions and interventions for dimensional, rather than categorical, forms of PDs in young people. Randomised controlled trials are needed to establish a more robust evidence base for effective treatments.

**Supplementary information:**

The online version contains supplementary material available at https://doi.org/10.1007/s00787-026-03030-6.

## Introduction

 Personality disorders (PDs) are a mental health condition that impacts a person’s cognitions, emotions, behaviours, interpersonal relationships and sense of self [1]. Diagnostic systems such as the DSM and ICD have recognised that personality disorder features may be identifiable before age 18 when they are sufficiently pervasive and persistent (with the DSM-5 exception that antisocial personality disorder cannot be diagnosed prior to 18 years). In clinical and research contexts, such early presentations are often described as **“**emerging PDs”. A life span and developmental perspective model suggests that emerging PD occurs during a vulnerable risk factor period [2, 3]. Across studies, the prevalence of personality disorders in adolescent community samples and primary care settings typically falls between 6% and 17% [4, 5]. PDs in young people are associated with impaired intra- and interpersonal functioning [6, 7], lower educational achievements [7, 8], and poorer physical, mental, and sexual health [7, 9].

Despite the link between emerging PDs and various mental, social, and physical health impairments [10–13], diagnosis in young people is often delayed [14, 15] and access to treatment is often limited [16]. For instance, in Europe, a study reports 57.8% of psychologists across Europe recognise emerging PDs, yet only 6.5% offer treatment aimed at improving PD-related impairments [14]. Delay in diagnosis and treatment are often driven by stigma and discrimination [17], limited awareness, identification and assessment tools for young people [18, 19], and ongoing debate about appropriateness of diagnoses [20] due to concerns about whether it is developmentally appropriate and psychometrically sound to apply categorical PD labels before personality is fully consolidated. Delay in diagnosis and treatment of PD in young people may be clinically harmful and lead to worse symptoms and outcomes [18, 21]. Empirical evidence shows early treatment can lead to improvements in personality functioning over time including significant reductions in PD diagnoses and trait changes [22].

Delay in treatment could also be related to conflicting research regarding efficacy of different treatment modalities. Currently, there is no systematically reviewed evidence guiding the use of/efficacy of psychological, social and pharmacological prevention and intervention for young people with emerging, subthreshold or diagnosed PDs. Previous systematic reviews have primarily focused on treatments for PDs in adult populations, with most studies targeting specific subtypes, such as BPD [23]. Only a few reviews have examined interventions for emerging BPD [24–26]. A meta-analysis by Jørgensen et al. (2021) identified only 10 trials of psychological therapies for adolescents with BPD or BPD features, with few showing clear superiority over controls and no reported adverse effects. Evidence quality was low due to high risk of bias, small samples, and high attrition, making it difficult to draw firm conclusions and highlighting the need for larger, high-quality trials [24]. A narrative review examining pharmacological treatments for BPD in adolescents showed low-certainty evidence [25], and a 2022 Cochrane review indicated little to no effect of antipsychotics, antidepressants, or mood stabilisers on core BPD symptoms compared to placebo, and guidelines recommend psychotropic drugs only for co-occurring conditions or acute crises [27]. Although this Cochrane review included people under the age of 25 years and adults, all results were pooled across the age range and no age-stratified subgroup analyses were performed.

While prior reviews have focused on adults and on BPD specifically, the intervention landscape for young people with emerging personality pathology is broader and increasingly informed by dimensional conceptualisations. Yet no comprehensive overview has collated psychological, psychosocial, pharmacological, and preventive approaches developed for youth, or documents their targets, delivery characteristics, and outcomes across personality presentations. By mapping this terrain, our review provides an informed picture of what is available now and identifies where evidence is lacking or too fragmented to support firm conclusions, thereby setting a clear agenda for future, focused effectiveness research.

## Methodology

### Literature search

We followed the Preferred Reporting Items for Systematic Reviews (PRISMA) guidelines (Supplementary Material 1). The protocol for this study was pre-registered on PROSPERO (CRD42020202035).

**Fig. 1:**
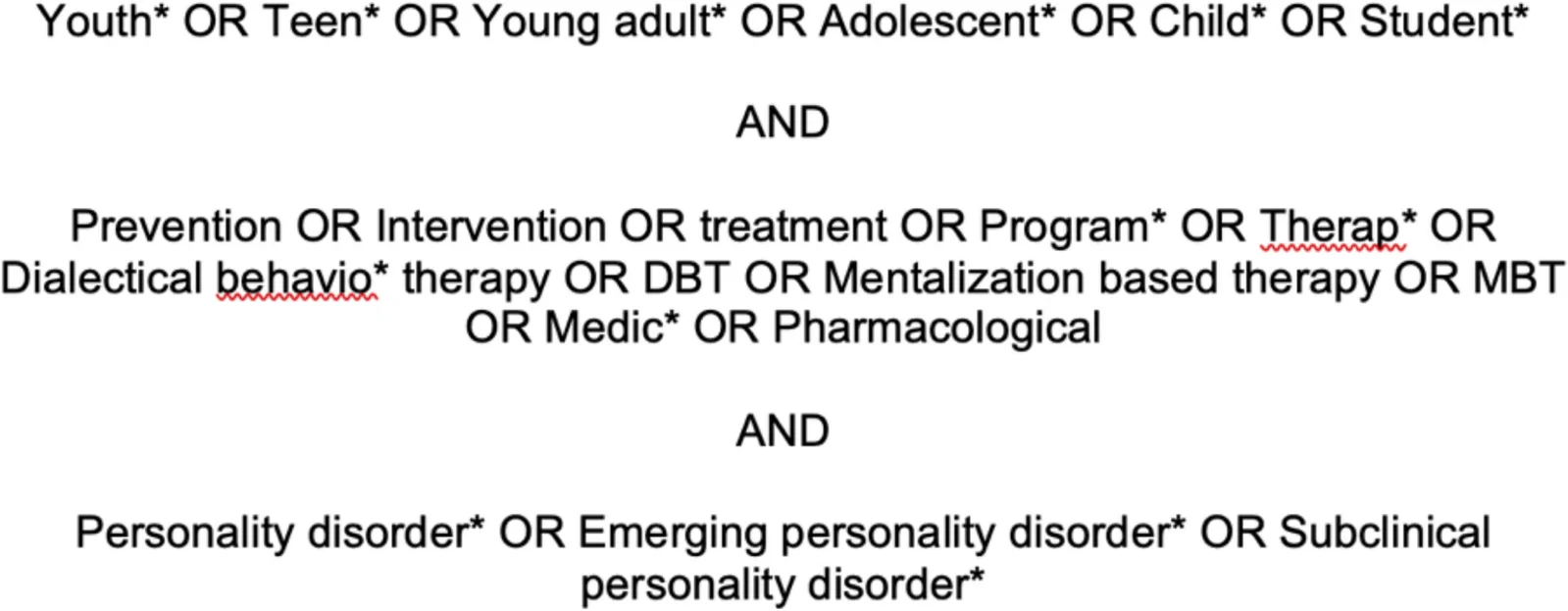
Search terms

We searched four electronic databases: Medline, PsycINFO, Embase and Web of Science. The initial search combined three concepts, following the PICO structure: (1) young people, (2) prevention and intervention and (3) personality disorders (Fig. 1). Grey literature, such as dissertations, was included when indexed within these databases. We consulted with a clinical psychologist working with young people with PDs and a person with lived experience of a PD on the relevance and appropriateness of search terms and protocol.

MeSH terms were used in conjunction with text words to enhance search results. Alongside broad psychotherapy terms (e.g., therap*, intervention*, *treatment*, program*), we included DBT and MBT as named terms because they are widely implemented and researched in young people with emerging personality pathology [28–30] and were recommended by the clinical psychologist and service user we consulted with.

The original search was conducted in July 2020, and was updated in May 2024.

### Inclusion criteria

Studies were eligible if they included young people up to and including 25 years old, who had a subthreshold or threshold PD diagnosis, with the term ‘emerging PD’ used only in relation to adolescents, as identified by clinical judgement, ICD or DSM criteria. There was no lower age limit. The upper age limit reflects those included in youth mental health services in countries such as Australia and the United Kingdom [31], as well as contemporary research that suggest adolescence extends into the mid-20s [32]. For studies with a sample of young people and adults, the studies were included if 50% or more of participants were up to the age of 25 years old or if the data was aggregated according to age. Where the data were unclear about age, we contacted authors for clarification. We included studies with comorbid mental health disorders if the relevant data could be extracted. Quantitative and qualitative studies in English that examined psychosocial, psychological or pharmacological preventions or interventions were included in the review. Prevention is defined as a treatment that seeks to reduce or eliminate the likelihood of a mental health condition, such as PD, from development and persistence over time [33, 34]. An intervention is defined as a treatment that seeks to treat a mental health condition, such as PD, after its onset [35]. Studies were excluded from the review if (1) the preventions or interventions were targeted towards carers, family, friends, teachers, or mental health professionals, (2) the main context of the study focused on a specific service, such as hospitals, without investigating a preventions or interventions for the target population, (3) the study was not published in English, (4) systematic reviews, meta-analysis, case studies or narrative general reviews or (5) if the study was found to produce a low quality rating. Low-quality studies were excluded because they present a high risk of bias and methodological weaknesses that could undermine the reliability of the review’s conclusions.

#### Scope clarifications in line with PROSPERO

Our PROSPERO protocol specified a focus on interventions developed for or applied directly to emerging or threshold personality pathology. To maintain this focus, we interpreted eligibility as interventions where young people were recipients and primary outcome targets. This approach excluded programmes aimed solely at caregivers, family, friends, teachers or professionals, or service-level changes, as these represented distinct mechanisms and contexts. Studies set exclusively in acute hospital or emergency settings were considered outside scope when they evaluated service configuration rather than a defined clinical intervention for the young person.

### Study selection

All titles and abstracts were deduplicated and screened by two reviewers (RM and KP) in Covidence, a systematic review screening and extraction platform [36]. This represents a deviation from the pre-registered protocol, which stated that only 20% would be double-screened. Full-texts of all studies were then screened by two reviewers (RM and KP) following inclusion and exclusion criteria. Agreement was moderate for full-text review (interrater agreement Cohen’s kappa = 0.734). Disagreements at any stage between reviewers were discussed and resolved by a third reviewer (SR).

### Data extraction and quality assessment

The following characteristics were extracted from the included studies: author, country, sample (including the emerging PD, amount of female and male participants and age range), setting, study design, prevention/intervention, primary outcome measures and results. The data was extracted by one reviewer (RM) and a second reviewer (KP) conducted an independent data extraction of 50% of included studies.

Quality assessment of all studies was assessed by one reviewer (RM) and a second reviewer (KP) assessed 50% of included studies. Discrepancies were resolved through discussion, and good consensus was reached which provided a reliability check. We did not calculate inter-rater agreement, as this is not standard practice when only a subset of studies is double-rated. Effective Public Health Practice Project (EPHPP) [37], a six-item tool, was chosen to assess quality of quantitative studies. Studies were rated as high, moderate, or low quality their scores across six domains (selection bias, study design, confounders, blinding, data collection method and withdrawals and dropouts). Studies were rated as low quality if they received low ratings in more than one section. Critical Appraisal Skills Programme (CASP) tool [38] was selected to assess the quality of qualitative studies. While CASP does not generate a formal quality rating, it guides appraisal through answered 10 questions answered with “yes”, “no”, or “can’t tell”. The more questions that received a “yes” response, the higher the study was judged in quality.

### Data synthesis

A meta-analysis was not conducted due to substantial heterogeneity in study designs, interventions, and outcomes. As the review focused on identifying the range of available treatments rather than evaluating effectiveness, a narrative synthesis was more appropriate.

A narrative synthesis was conducted following Popay et al.‘s (2006) [39] guidelines for integrating quantitative and qualitative evidence. A preliminary framework was developed to examine how different prevention and intervention approaches might improve outcomes for emerging PDs and reduce long-term negative consequences. Studies were grouped by intervention type, and key characteristics and findings were tabulated. Outcomes were described narratively, focusing on patterns across studies. Outcomes included PD symptoms, self-harm, suicidal ideation and attempts, internalising and externalising symptoms, depression, anxiety, hospital and emergency service use, quality of life, dropout, and adverse events. These findings were then compared across studies to provide an overall picture of outcomes associated with each intervention type.

## Results

**Fig. 2:**
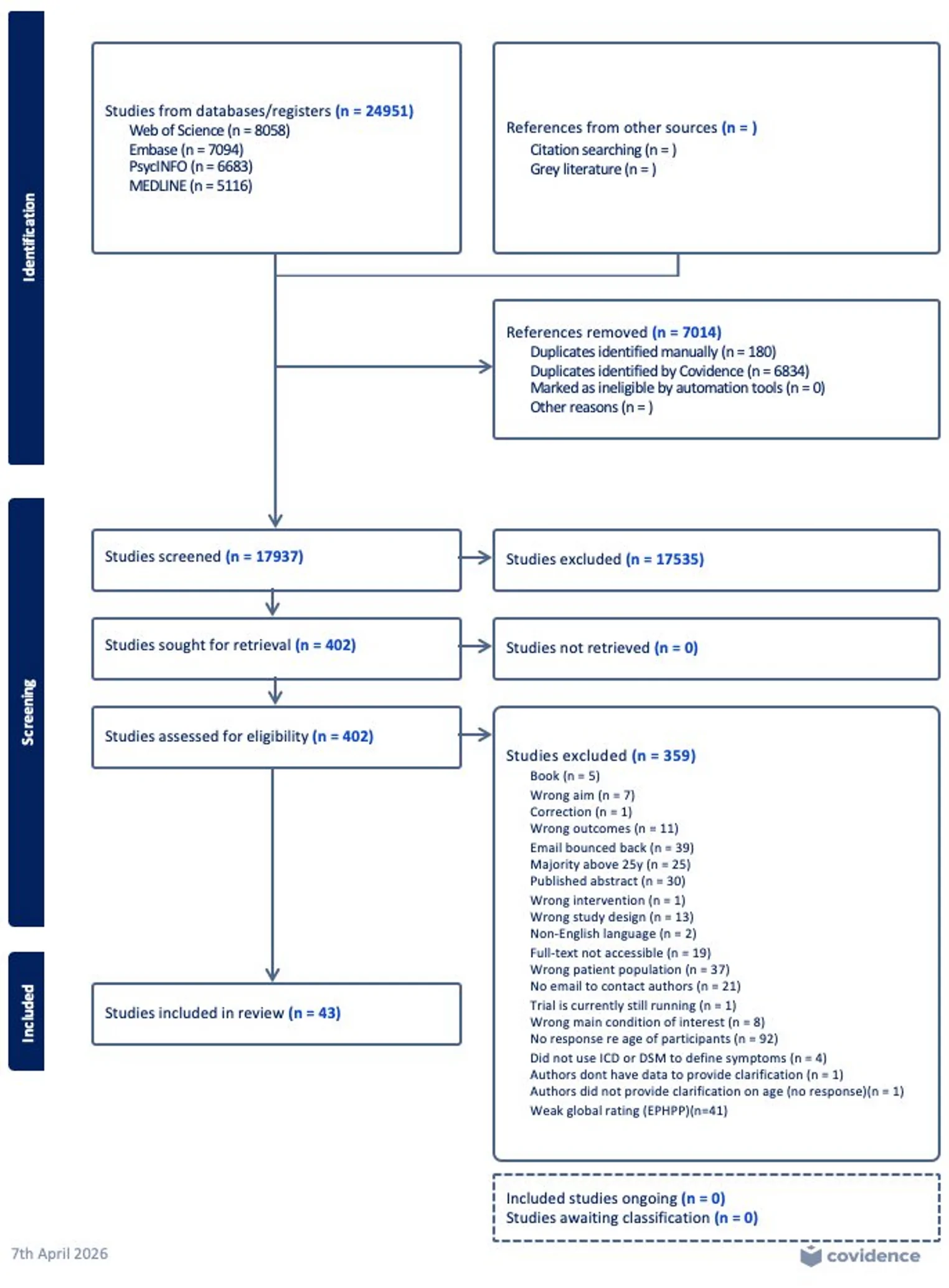
PRISMA diagram

After screening titles and abstracts, 402 studies were considered eligible for full text examination. Following full text examination, 43 studies were included in this study (Fig. 2).

### Study characteristics

Study characteristics can be found in Tables 1, 2 and 3. Studies were published from 2008 to 2024, with over half (*n* = 23) of studies published between 2019 and 2024. Twenty-one studies were conducted in Europe, seven in the United States of America, six in Australia, three in the United Kingdom, one in India, and one in Iran. One study did not indicate the country [40] and one study included multiple locations (Germany, Chile, and Switzerland) [41].

**Table 1:** Summary of psychological preventions/interventions

Author	Country	Sample	PD Assessment	Setting	Study Design	Prevention/Intervention	Results
Andersen et al. (2021)	N/A	BPD traits Female: 55 Total: 111 14–17 years	Interview: CI-BPD	Natural Clinical Setting	Qualitative	Intervention MBT-G Follow-up: End of treatment	*Five Themes*: Negative experiences Incorrect treatment Difficulty with group format Changes in group make-up and treatment course Experiencing negative emotions
Bartsch et al. (2024)	Australia	BPD symptoms Female: 105 Total: 109 16–18 years	Self-report: MSI-BPD, BSL-23/-supp	Community/Outpatient	Acceptability & Feasibility	Intervention Road Maps Follow-up: End of treatment	Emergency department visits decreased 6-months post treatment Non-significant decrease in inpatient admissions and rate of bed days
Beck et al. (2020)	Denmark	BPD criteria Female: 111 Total: 111 14–17 years	Interview: MINI-KID 6.0, CI-BPD, SCID-II	Outpatient	RCT	Intervention MBT-G/TAU Follow-up: End of treatment	MBT-G and TAU: BPD features produced same results Increase in hospital and emergency room visits
Bo et al. (2019)	Denmark	AVPD pathology Female: 7 Total: 8 14–17 years	Interview: SCID-II Self-report: MACI	Child & Adolescent Psychiatric Clinic	Pre/post	Intervention MBT-G Follow-up: End of treatment	Significant decrease in AVPD and internalising scores Non-significant decrease for externalising scores
Boon et al. (2022)	Netherlands	PDs Female: 101 Total: 116 14–22 years	Self-report: VKP Interview: SCID-II	Partial residential psychotherapeutic facility	Pre/post	Intervention CERS Follow-up: End of treatment	Significant decrease in PD symptoms
Buerger et al. (2019)	Germany	BPD traits Female: 66 Male: 6 Total: 7212-17 years	Interview: SCID-II	Outpatient	Single group pre/post	Intervention DBT-A + GCC Follow-up: 25-weeks	Decrease in BPD criteria for diagnosis and suicide attempts
Chanen et al. (2008)	Australia	BPD subsyndromal/ full-syndrome Total: 7815-18 years	Interview: SCID-I/P, SCID-II, K-SADS-PL	Outpatient HYPE Programme	RCT	Intervention CAT/GCC Follow-up: 24-months	CAT and GCC: Decrease found in parasuicidal behaviour CAT: Decrease in externalising symptoms
Chanen et al. (2009)	Australia	BPD subsyndromal or full-syndrome Female: 82 Total: 110 15-18 years	Interview: SCID-I/P, SCID-II, K-SADS-PL	HYPE Programme	Quasi-experimental	Intervention CAT/ H-TAU/GCC: Follow-up: 24-months	All interventions: Decrease in parasuicidal behaviour CAT: Decrease in internalising and externalising symptoms
Chanen et al. (2022)	Australia	BPD diagnosis Female: 104 Male: 24 Total: 128 15-25 years	Interview: SCID-II	HYPE Programme	RCT	Intervention HYPE+CAT vs. Befriending/ HYPE vs. YMHS+Befriending Follow-up: 18-months	HYPE+CAT and HYPE: no significant decrease in interpersonal problems, social adjustment and suicidal ideation at 12-months
Courtney & Flament (2015)	Canada	BPD diagnosis Female: 57 Male: 4 Total: 61 15-18 years	Assessed by a psychiatrist (DSM-IV diagnosis)	Youth Programme of the Royal Ottawa Mental Health Center	Open-label naturalistic study	Intervention A-DBT-AFollow-up: End of treatment	Significant decrease in suicidal ideation, PD symptoms and self-harm
Denaro (2008)	USA	BPD features Female: 19 Total: 19 14-17 years	Interview: SCID-II	Outpatient	Cohort analytic (2 groups)	Intervention DBT/TAU Follow-up: End of treatment	Significant decrease in suicidal ideation and depression symptoms Non-significant decrease in parasuicidal behaviour, anxiety and anger
Feenstra et al. (2014)	Netherlands	AVPD, OCPD, BPD, PDNOS, Any PD pathology Female: 58 Male: 12 Total: 70 14-19 years	Interview: SCID-II	Inpatient	Descriptive Study	Intervention IPA Follow-up: 6, 12, 24-months	Significant decrease in symptom severity
Gleeson et al. (2012)	Australia	BPD features (four or more) Male: 3 Total: 16 Mean age: 18.4 years	Interview: SCID-I/P, SCID-II	Orygen Youth Health (OYH)	Pilot RCT	Intervention HYPE+SFET/SFETFollow-up: 6-months	Non-significant decrease in psychotic symptoms, functioning and irritability
Hauber et al. (2017)	Netherlands	PD traits and symptoms Female: 93 Total: 9715-22 years	Self-report: VKPInterview: SCID-II	Residential psychotherapeutic institution	Prospective pilot study	Intervention MBT Follow-up: End of treatment	Significant decrease in PD criteria and symptoms
Hauber et al. (2020)	Netherlands	PDs Female: 50 Male: 10 Total: 67 15-22 years	Clinical diagnosis (DSM-III)	Partial residential psychiatry institution	Observational, cross-sectional, prospective	Intervention MBT-A Follow-up: End of treatment	Decrease in psychological distress
Jørgensen et al. (2024)	Denmark	BPD diagnosis (above clinical cut-off) Female: 96 Total: 97 19-23 years	Interview: SCID-5-PD	Mental health services	RCT	Intervention MBT-G+TAU Follow-up: End of treatment	24% of participants met criteria for BPD diagnosis
Lakeman & Emeleus (2020)	Australia	BPD diagnosisTotal: 617.4-25.3 years	Self-report: BSL-23	DBT programme for youth	Qualitative	Intervention DBT: weekly individual therapy sessions, 20-week skills group 3 hours, phone coaching when necessary, total 12-weeks of attendance. Follow-up: 3-6 months.	Three Themes: DBT cheerleader DBT language and consolidation of skills Elements of success
Lind et al. (2023)	Denmark	BPD or subthreshold BPD (at-least 4 BPD criteria) Female: 6 Total: 6 14-16 years	Interview: CI-BPD	Department of Child and Adolescent Psychiatry	Qualitative	Intervention MBT-G: 37 weekly 90 minute sessions. Follow-up: End of treatment.	Two Themes: Agency Communion
Lyng et al. 2020	Ireland	BPD diagnosis Female: 29 Male: 8 Total: 37 18-56 years (subgroup: 18-25 years)	BPD diagnosis by a psychiatrist Self-report: BSL-23	Community adult mental health centers	Naturally occurring quasi-experimental design	Intervention General adult DBT/Young adult only DBT Follow-up: End of treatment	Young adult only DBT Program: Decrease and large effect size (15-16%) in BPD symptoms at post-treatment
Mehlum et al. (2016)	Norway	BPD (at-least 2 criteria from DSM-IV or 1 criteria + subthreshold-level criteria) Female: 68 Male: 9Total: 77 Mean age 15.6 years	BPD symptoms using DSM-IV Self-report: BSL-23	Outpatient	Prospective follow-up	Intervention DBT-A/EUC Follow-up: 19 and 71-weeks (1 year follow-up).	DBT-A: No suicidal attempts and decrease in self-harm and borderline symptoms at follow-up
Mohamadpour et al. (2024)	Iran	BPD traits Female: 0 Total: 4012-18 years	Confirmation of disorder by psychologist Self-report: BPFS-C	Online	Quasi-experimental pre-test-post-test	Intervention Online integrative trans-diagnostic treatment/Waitlist Follow-up: 3-months	Significant decrease between groups for internet addiction and high-risk behaviours at end of treatment Decrease in BPD scores at follow-up
Moran et al. (2018)	USA	BPD criteria or diagnosis Female: 53 Total: 53 13-20 years	Interview: SCID-II with confirmation by primary psychiatrist or psychotherapistSelf-report: ZAN-BPD	Residential program	Single pre/post	Intervention DBT-residential programme Follow-up: End of treatment	Significant decrease in depressive and BPD symptoms after 1-month of treatment Non-significant decrease in anxiety scores after 1-month of treatment
Ohlis et al. (2023)	Sweden	Self-harming + traits of BPD (at least four 4/9 criteria) Female: 18 Male: 1 Total: 19 <18 years of age at the start of treatment	At least four of nine diagnostic criteria for BPD, diagnostic assessment N/S	Child and Mental Health Services	Qualitative	Intervention DBT-AFollow-up: Endpoint	Six Themes: Need to be seen, listened and believed in Importance of teamwork (patient and therapist) Value of group/structure Therapy as life-saving and life-changing Risks of feeling misplaced Risks of abrupt endings
Phillips et al. (2024)	UK	Emerging BPD/ASD DisorderFemale: 235 Male: 7 Total: 242 13-17 years	Interview: SCID-BPD Self-report: MSI-BPD	Child and Mental Health Services	Pre/post	Intervention DBT Follow-up: End of treatment	Significant reduction in BPD and emotion dysregulation scores for participants with ASD Non-significant reduction for self-harm, inpatient days and A&E for both groups (with and without ASD)
Pistorello et al. (2012)	USA	NSSI and BPD (3 or more criteria) Female: 51 Total: 63 18-25 years	Interview: SCID-II	College	RCT	Intervention DBT/O-TAUFollow-up: 18-months	Significant reduction in suicidality (during treatment and at follow-up), depression (at 6, 12 and 18-months follow-up) and NSSI No decrease in BPD diagnosis at follow-up
Popolo et al. (2018)	Italy	AVPD, DPD, OCPD, NPD, PPD, Passive-Aggressive PD, Depressive PD; PDNOS (at least 10 criteria) Female: 14 Total: 17 16-23 years	Interview: SCID-11	Outpatient	Feasibility	Intervention MIT-G Follow-up: 3-months	Large effect size between pre and post treatment for clinical outcomes, emotion regulation, impulse control and emotional awareness Significant reduction in alexithymia Low dropout rate (2/17)
Popolo et al. (2019)	Italy	AVPD, DPD, OCPD, NPD, PPD, Passive-Aggressive PD, Depressive PD criteria; PDNOS (at least 10 criteria) Female: 9 Total: 2019-25 years	Interview: SCID-II	Outpatient	RCT	Intervention MIT-G/Waitlist + TAU Follow-up: 3-months	MIT-G Significant decrease in clinical outcomes scores Attendance rate = 92.19%
Popolo et al. (2022)	Italy	AVPD, DPD, OCPD, NPD, PDNOS, Depressive PD, Passive-Aggressive; PDNOS (at least 10 criteria) Female: 27 Male: 13 Total: 40 18-25 years	Interview: SCID-II Meeting DSM-IV diagnosis	Outpatient	RCT	Intervention MIT-G/Waitlist + TAU Follow-up: 3-months	MIT-G: Decrease in clinical outcomes from baseline to follow-up; did not remain significant at follow-up 100% completion rate
Rockstroh et al. (2023)	Germany	NSSI and BPD pathology and diagnostic criteria Female: 71 Total: 74 14.9 years mean age	Interview: SCID-II	Clinic of Child and Adolescent Psychiatry at the University Hospital	Prospective cohort group follow-up	Intervention CDP/TAU Follow-up: End of treatment	Significant decrease in NSSI from T2 to T3 Significant reduction in suicide attempts (T0-T2, T2-T3) but not at T3 Significant decrease in BPD scores from T0-T3
Rossouw & Fonagy, (2012)	UK	Self-harm and BPD Female: 68 Total: 80 14.7 years mean age	Interview: CI-BPD	Community mental health services or acute hospital emergency rooms	RCT	Intervention MBT-A/TAU Follow-up: 3, 6, 9 and 12-months	Significant decrease in self-harm, depression, BPD features at 12-months 33% met criteria for BPD diagnosis at 12-months following MBT-A
Salzer et al. (2014)	Germany	BPD diagnostic criteria + F-92 ICD-10 diagnosis (applicable) Female: 22 Total: 28 14-19 years	Interview: SCID-I, SCID-II Structured Diagnostic System: DISYPS-KJ	Outpatient	Pre/post	Intervention Psychodynamic therapy Follow-up: End of treatment	After treatment: 50% of patients were below the cut-off score for BPD
Schiffler et al. (2022)	Austria	BPD diagnosis Female: 12 Male: 1 Total: 13 18-23 years	Diagnosis via ICD-10	Psychiatric ward, hospital	Qualitative	Intervention Mobile DBT App Follow-up: End of treatment	Five themes: Experience with DBT skills Phenomenon of self-harm Feelings about self-harm Dealing with disordered-specific symptoms Prevention of self-harm
Schmeck et al. (2023)	Switzerland, Germany and Chile	BPD (full or subthreshold) Female: 56 Total: 60 13-19 years	Interview: SCID-II	Hospital	Non-RCT (non-inferiority design)	Intervention AIT/DBT-A Follow-up: End of treatment	AIT group: Significant decrease in depression Suicide attempts: Non-significant improvements
Schuppert et al. (2009)	Netherlands	BPD traits/symptoms /clinical/subclinical Female: 38Total: 4314-19 years	Interview: BPDSI-IV	Mental health centers	RCT	Intervention ERT/TAU Follow-up: Post treatment	Internalising scores: Significant improvements BPD severity and externalising scores: Non-significant improvements
Schuppert et al. (2012)	Netherlands	BPD features Female: 105 Total: 109 14-19 years	Interview: SCID-II, BPDSI-IV-ado	Mental health centers	RCT	Intervention ERT + TAU/TAU Follow-up: Post treatment	Non-significant changes: Any of the measurements between groups
Simonsen et al. (2021)	Denmark	BPD (at least 4 DSM-5 criteria or score above clinical cutoff on BPFS-C) Female: 96 Male: 1 Total: 96 (at follow-up) 16.25 years mean age	Interview: SCID-II, MINI-KID 6.0 Self-report: BPFS-C	Outpatient	RCT	Intervention MBT-G/TAU Follow-up: 2 years	No difference in BPD symptoms or remission at follow-up between groups
Vijayapriya & Tamarana (2023)	India	BPD diagnosis/BPD traits Female: 25 Male: 11 Total: 36 18-25 years	Self-report: MCMI-III (diagnosis or confirmation by a licensed clinical psychologist)	College	Non-RCT	Intervention Internet Delivered DBT Skills Training/Behavioural Activation Follow-up: End of treatment	DBT: Significant difference in executive functioning scores

*USA* united states of america, *UK* united kingdom, *PDs* personality disorders, *BPD* borderline personality disorder, *AVPD* avoidant personality disorder, *OCPD* obsessive-compulsive personality disorder, *NPD* narcissistic personality disorder, *PPD* paranoid personality disorder, PDNOS personality disorder not otherwise specified, *NSSI* non-suicidal self-injury, *DBT* dialectical behaviour therapy, *DBT-A* dialectical behaviour therapy for adolescents, *MBT* mentalisation based therapy, *MBT-G* group mentalisation based therapy, *CAT* cognitive analytic therapy, *ERT* emotion regulation training, *MIT-G*group metacognitive interpersonal therapy, *IPA* inpatient psychotherapy for adolescents, *CERS* cognitive emotion regulation strategies,*CDP* cutting down programme, *HYPE* helping young people early, *YMHS* youth mental health service, *SFET* specialist first-episode psychosis treatment, *AIT* adolescent identity treatment, *TAU* treatment-as-usual, *H-TAU* historical treatment-as-usual, *EUC* enhanced usual care, *GCC* good clinical care, *RCT* randomised controlled trial, *MSI-BPD* mclean screening instrument for borderline personality disorder, *BSL* borderline symptom list, *MINI-KID* mini-international neuropsychiatric interview for children and adolescence, *CI-BPD* childhood interview for dsm-iv borderline personality disorder, *SCID-I/P* structured clinical interview for *DSM-IV* axis I disorders – patient version, *SCID-II* structured clinical interview for dsm-iv-axis II, SCID-5-PD structured clinical interview for dsm-5 personality disorders, *MACI* million adolescent clinical inventory, VKP dutch questionnaire for personality characteristics, *K-SADS-PL* kiddie schedule for affective disorders and schizophrenia for school-age children – present and lifetime version, *DISYPS-KJ* diagnostic checklist for conduct disorders of the diagnostic system for mental disorders in childhood and adolescence, *BPDSI-IV* borderline personality severity Index-IV, *MCMI-III* million clinical multiaxial inventory-III,  *N/S* not specified, *ASD* Autism Spectrum Disorder

**Table 2:** Summary of psychosocial preventions/interventions

Author	Country	Sample	PD Assessment	Setting	Study Design	Prevention/Intervention	Results
Kellam et al. (2008)	USA	First grade students, ASPD rates Female: 462 Male: 460 Total: 922 19–21 years at follow-up	Psychiatric Interview: CIDI-UM	Five urban areas of Baltimore City, and matching sets of schools in each area	Epidemiologically Based, Randomised Field Trial Design	Preventative Intervention GBG Follow-up: At ages 19–21 years	GBG: Decrease in drug use/dependence, alcohol use/dependence, antisocial PD and depression scores No difference between all 3 conditions for anxiety
Petras et al. (2008)	USA	First grade students, ASPD prevalence Male: 199 (first cohort), 181 (second cohort) Total: 768 19–21 years at follow-up	A scale was created to assess for ASPD and conduct disorder via DSM-IV (scale name N/S)	Baltimore city public schools	RCT	Preventative Intervention GBG Follow-up: At ages 19–21 years	Significant decrease in antisocial PD diagnosis for males in the persistently high class: 40.1% after GBG Non-significant decrease in antisocial PD diagnosis for females in the persistently high and medium class
Scott et al. (2014)	UK	ODD symptoms and diagnosis, antisocial behaviour and ASPD traits Male: 90 (indicated) + 76 (early)(at entry)Total: 120 (indicated) + 684 (selective) = 8043-7 years and 4-6 years	Investigator based interview: Parent Account of Child SymptomsInterview: Child and Adolescent Psychiatric AssessmentParent reports: Antisocial Process Screening DeviceSelf-report: Strengths and Difficulties Questionnaires for Parents and Teachers (conduct problem scale)Self-Report: Self-Report Delinquency instrument	Child mental health clinics/school setting	Long-term follow-up	Preventative Intervention The Incredible Years Programme1. Indicated Early Intervention Study: Average of 9 sessions.2. Selective High-Risk Early Intervention Study: Average of 15 sessions.Follow-up: At ages 4-10 years	Treatment in early childhood: decrease of ODD and ASPD trait rates No long-term results for the selective high-risk population

*USA* united states of america, *UK* united kingdom, *RCT* randomised controlled trial, *GBG* good behaviour game, *PD* personality disorder, *ODD* Oppositional Defiant Disorder, *ASPD* Antisocial Personality Disorder, *MDD* major depressive disorder,  *N/S* not specified

**Table 3:** Summary of pharmacological preventions/interventions

Author	Country	Sample	PD Assessment	Setting	Study Design	Prevention/Intervention	Results
Amminger et al. (2013)	Austria	BPD diagnosis and Ultra-High-Risk Criteria for Psychosis Female: 14 Total: 15 13–25 years	Diagnosis by two psychiatrists and one clinical psychologist (DSM-IV)	Medical University Department	RCT	Intervention Omega-3 Fatty Acid Supplementation/Coconut Oil Follow-up: End of treatment (12-weeks)	Significant decrease in BPD and depression symptoms at end of treatment compared to placebo
Ha (2017)	USA	BPD full diagnosis Female: 31 Total: 40 12–17 years	Interview: CI-BPD	Inpatient	RCT	Intervention Intranasal Oxytocin/Placebo Nasal spray Follow-up: End of treatment	No significant outcomes for decrease in mentalisation
Raine et al. (2024)	USA	SPD scores Female: 84 Male: 94 Total: 290 11–12 years	Self-report: SPQ-C	N/S	RCT	Prevention CBT/Omega-3/Omega-3 + CBT/Control Follow-up: 3, 6, 12-months	Omega-3 and Omega-3 + CBT decreased schizotypy at baseline and 3 months follow-up Omega-3 and Omega-3 + CBT decreased interpersonal scores related to schizotypy No significant decrease in cognitive perceptual scores

*BPD* borderline personality disorder, *SPD* schizotypal personality disorder, *USA* united stated of america, *RCT* randomised controlled trial, *CBT* cognitive behaviour therapy, *N/S* not specified, *DSM-IV* Diagnostic and Statistical Manual of Mental Disorders-IV, *CI-BPD* childhood interview for dsm-iv borderline personality disorder, *SPQ-C* schizotypal personality questionnaire-child, *DSM-IV* Diagnostic and Statistical Manual of Mental Disorders-IV

Sixteen studies included participants aged 18 years and under, four studies included only young adults aged 19 to 25 years, eighteen studies included a mixed sample of adolescents and young adults and five provided mean age data. Majority of studies (*n* = 39) examined an intervention approach, while only four studies examined a preventative approach.

### Quality assessment

Quality assessment ratings are presented in Tables 4 and 5. Eighty-five studies were assessed using the EPHPP or CASP tool. Thirty-eight quantitative studies received high (29%) or moderate (due to receiving a weak score in one category) (71%) ratings using EPHPP. The remaining 41 quantitative studies were found by EPHPP to be of low quality and were excluded, as per our eligibility criteria. All five qualitative studies assessed by CASP reflected valid results, appropriate methodology, research design, and data collection which clearly addressed the research question, ethical considerations, rigorous data analysis and overall valuable research. One qualitative study did not provide enough information on the reason for selecting participants, discussion on why some participants could not take part in the study and was primarily skewed only to positive experiences [42].

**Table 4:** Assessment of risk of bias of quantitative studies: EPHPP tool

Study	Selection Bias	Study Design	Confounders	Blinding	Data Collection Methods	Withdrawals/drop outs	Global Rating
Randomised Controlled Trial							
Amminger et al. (2013)	M	S	M	S	S	S	S
Beck et al. (2020)	M	S	M	M	S	W	M
Chanen et al. (2008)	M	S	S	M	S	W	M
Chanen et al. (2022)	S	S	M	W	S	S	M
Gleeson et al. (2012)	S	S	S	W	S	S	M
Ha (2017)	M	S	S	S	S	S	S
Jørgensen et al. (2024)	S	S	S	W	S	S	M
Pistorello et al. (2012)	M	S	S	M	S	W	M
Popolo et al. (2019)	M	S	S	S	S	S	S
Popolo et al. (2022)	M	S	S	S	S	S	S
Raine et al. (2024)	M	S	S	M	M	W	M
Rossouw & Fonagy (2012)	S	S	S	S	S	S	S
Schuppert et al. (2009)	S	S	S	M	S	M	S
Schuppert et al. (2012)	S	S	S	M	S	S	S
Simonsen et al. (2021)	M	M	S	W	S	S	M
Cohort (one group pre + post)							
Bo et al. (2019)	M	M	S	W	S	S	M
Boon et al. (2022)	M	M	S	W	S	M	M
Buerger et al. (2019)	M	M	M	W	S	S	M
Moran et al. (2018)	M	M	M	W	S	S	M
Salzer et al. (2014)	M	M	M	W	S	M	M
Non-Randomised Controlled Trial							
Schmeck et al. (2023)	M	M	M	W	S	M	M
Vijayapriya et al. (2023)	M	M	S	W	S	M	M
Cohort Analytic (two group pre and post)							
Denaro (2008)	M	M	S	W	S	M	M
Phillips et al. (2024)	M	M	M	W	S	M	M
Other: Prospective follow-up study							
Mehlum et al. (2016)	S	M	S	M	S	S	S
Rockstroh et al. (2023)	M	M	S	M	S	S	S
S, Strong (1); M, Medium (2); W, Weak (3)							
Other: Long-term follow-up							
Scott et al. (2014)	M	M	S	M	S	M	S
Other: Quasi-experimental study design							
Chanen et al. (2009)	M	M	S	S	S	S	S
Mohamadpour et al. (2024)	M	M	S	W	S	S	M
Lyng et al. (2020)	M	M	S	W	S	S	M
Other: Open Label Naturalistic Study							
Courtney & Flament (2015)	M	M	S	W	S	M	M
Other: Feasibility study							
Bartsch et al. (2024)	M	M	M	W	S	S	M
Popolo et al. (2018)	M	M	W	M	S	S	M
Other: Descriptive Study							
Feenstra et al. (2014)	M	M	M	W	S	M	M
Other: Observational, Cross-sectional and Prospective							
Hauber et al. (2020)	M	M	S	W	S	S	M
Other: Prospective Pilot Study							
Hauber et al. (2017)	M	M	S	M	S	W	M
Other: Randomised Field Trial Design							
Kellam et al. (2008)	S	M	S	W	S	S	M
Petras et al. (2008)	S	M	M	W	S	M	M
S, Strong (1); M, Medium (2); W, Weak (3)							

Additionally, two studies did not provide sufficient information on the researcher’s role in the study and their potential biases [42, 43].

**Table 5:** Assessment of risk of bias of qualitative studies: CASP tool

	**Andersen et al. (2021)**	**Lakeman & Emeleus (2020)**	**Lind et al. (2023)**	**Ohlis et al. (2023)**	**Schiffler et al. (2022)**
Was there a clear statement of the aims of the research?	Y	Y	Y	Y	Y
Is a qualitative methodology appropriate?	Y	Y	Y	Y	Y
Was the research design appropriate to address the aims of the research?	Y	Y	Y	Y	Y
Was the recruitment strategy appropriate to the aims of the research?	Y	CT	Y	Y	Y
Was the data collected in a way that addressed the research issue?	Y	CT	Y	Y	Y
Has the relationship between researcher and participants been adequately considered?	Y	CT	N	Y	Y
Have ethical issues been taken into consideration?	Y	Y	Y	Y	Y
Was the data analysis sufficiently rigorous?	Y	Y	Y	Y	Y
Is there a clear statement of findings?	CT	N	Y	Y	Y
How valuable is the research?	CT	Y	Y	Y	Y
Yes, (Y); No, (N); Can’t Tell (CT)					

### Psychological preventions/Interventions

All studies which evaluated psychological treatment for young people with subthreshold or threshold personality disorder, including ‘emerging PD’ in adolescents, examined interventions; none examined prevention.

**Dialectical Behaviour Therapy (DBT)** Thirteen studies evaluated DBT (one-month to 12-months, 6–40 sessions), for young people with BPD, with follow-up from six-months to eight years. Study designs included one RCT, two non-RCTs, three pre-post one open-label naturalistic study, one cohort analytic with two groups, one naturally occurring quasi-experimental design, one prospective follow-up study and three qualitative studies.

Studies included weekly individual therapy, group skills training, and phone coaching. Others explored modified formats, such as internet-delivered or mobile app–based interventions. Only a minority evaluated the full DBT model [44–46].

Six quantitative studies reported statistically significant reductions in BPD symptoms/criteria at post-treatment/follow-up (p-values from 0.002 to < 0.01) [45–50]. Pistorello et al. (2012) found improvement during treatment (*p* = 0.0001) that was not maintained at follow-up [44]; Schmeck et al. (2023) also reported greater symptom reduction in the comparator (Adolescent Identity Treatment (AIT)) (*p* < 0.05) [41]. Majority emphasised p-values than standardised effect sizes, limiting comparisons of magnitude; where reported, effects were generally small-to-moderate. Findings suggest DBT may reduce BPD symptoms, but the long-term sustainability of these effects compared to other treatments remain uncertain due to mixed results and variability in protocols.

DBT’s impact on self-harm and suicidality was mixed. Of eight studies assessing self-harm, five reported significant reductions (*p* = 0.001–0.016) [41, 44, 46, 48, 49]; Denaro (2008) observed non-significant reductions over time (Score = 0, df = 1, *p* = 1.0) [51], and Schiffler et al. (2022) noted decreases may partly reflect factors beyond treatment (e.g., reduced desirability of scarring) [52]. Five studies evaluated suicidal ideation; four reported significant reductions (*p* < 0.001–0.022) [44, 48, 49, 51], whereas Lyng et al. (2020) did not [45]. This was potentially due to small sample size. Only Buerger et al. (2019) reported a significant reduction in suicide attempts at 6-month follow-up (*p* ≤ 0.001) [50]. Overall, reductions are more consistently observed for self-harm and ideation than for suicide attempts, and effect magnitude is difficult to compare across studies due to heterogeneous measures and reporting.

Results for co-occurring symptoms were variable. Three studies reported significant reductions in depressive symptoms (*p* < 0.001 – *p* = 0.004) following DBT at post-treatment and follow-up [44, 47, 51]. Three studies reported non-significant reduction in depressive symptoms at follow-up (*p* = 0.400–0.54) [48], in comparison to the comparator group (*p* = 0.052 for DBT versus *p* = 0.001 for AIT) [41] after applying Bonferroni corrections [46]. Anxiety was assessed by three studies and generally did not show significant change (*p* = 0.07–0.40) [46, 47, 51]. Given the limited and heterogeneous assessment of these domains, conclusions about transdiagnostic effects are tentative.

Completion rates were high in over half of studies (80% to 100%). However, limited evidence was found for reductions in inpatient bed days or emergency visits [46, 48].

None of the studies reported on presence or absence of adverse outcomes.

**Mentalisation Based Therapy (MBT)** Nine studies examined MBT as a treatment (37 sessions to one year duration) for young people with borderline and avoidant PD, encompassing three RCTs, one pre/post, two qualitative and one prospective study.

MBT was delivered as group-based, individual therapy sessions with adolescents, family therapy components, and intensive partial residential programmes. Only two studies included follow-up assessments (three-months to two years) [53, 54].

The outcome of MBT findings were mixed. One RCT reported significant reductions in BPD diagnosis (*p* < 0.05, d = 0.36), self-harm (decrease from 100% of participants to 56%), and depression (*p* < 0.04) [53]. Another found decreased internalising symptoms (*p* = 0.014) and PD severity (*p* < 0.001) in adolescents with avoidant PD [55], while a residential study reported traits (*p* = 0.000) and symptom (*p* = 0.000) improvements across a range of PDs [56]. Two studies found either comparable results [54], or no significant difference [57] between MBT in a group setting and treatment-as-usual (TAU) in reducing BPD features as well as increased use of hospital and emergency services [57]. Where remission or improvement rates were reported, these were broadly comparable across MBT and TAU.

Only one qualitative study [40] reported on presence of adverse outcomes, including increased negative emotional states and dissatisfaction with therapist engagement.

**Group Metacognitive Interpersonal Therapy (MIT-G)** Two RCTs and one feasibility study evaluated MIT-G (16-sessions) for young people with avoidant, dependent, obsessive-compulsive, narcissistic, paranoid, passive-aggressive, depressive PDs, and PDs not otherwise specified. All studies included follow-up assessments at three-months. The feasibility study explored MIT-G as a standalone intervention [58]. The RCTs compared it to waitlist combined with TAU [59, 60].

MIT-G was found to significantly reduce alexithymia (*p* = 0.021–0.0312) (which did not remain statistically significant at three-months follow-up) and improve clinical outcomes (*p* < 0.01) [59, 60]. The intervention demonstrated high acceptability, with completion rates of 80% and above. Both RCTs reported improvements in emotion regulation.

None of the studies reported on presence or absence of adverse outcomes.

**Cognitive Analytic Therapy (CAT)** Three RCTs and one quasi-experimental study examined CAT for young people with BPD. Two studies compared CAT to TAU or good clinical care involving 24 sessions [61, 62]. One study evaluated CAT with Helping Young People Early (HYPE) service model against a befriending psychotherapy control condition involving eight to 16 sessions [63]. Another study investigated HYPE, which incorporates a time-limited CAT approach, with specialist first episode psychosis treatment over 16 sessions [64]. Follow-up periods ranged from six to 24-months.

CAT was found to significantly reduce parasuicidal behaviour (*p* < 0.05, SD = 1.02), as well as improving externalising and internalising symptoms, compared to TAU (SD = − 0.69 for externalising, SD = -0.79 for internalising) and good clinical care (SD = -0.38 for externalising) [61, 62], although CAT did not always outperform good clinical care [61]. Moreover, Chanen et al. (2022) found befriending to be superior to CAT with HYPE in reducing suicidal ideation [63]. In contrast, HYPE with specialist first episode psychosis treatment was associated with reduced symptom severity, yet the sample size was too small to carry out significance testing, and it was linked to increased alcohol misuse [64]. No other adverse effects were reported.

**Emotion Regulation Training (ERT)** Two RCTs investigated 17 weeks of ERT for young people with BPD. One study investigated ERT versus TAU [65], the other investigated ERT combined with TAU compared to TAU [66]. Neither study included a follow-up measure. In both, no significant outcomes were identified except for a non-significant improvement in internalising symptoms following the ERT-only (*p* = 0.06) [65].

Neither study reported on presence or absence of adverse outcomes.

**Psychodynamic Therapy** One pre-post study explored psychodynamic therapy as an intervention for young people with BPD [67]. This study found a decrease in criteria (remission rate of 39.29%) and scores for BPD post-intervention (*p* = 0.006).

The study did not report on the presence or absence of adverse outcomes.

**Inpatient Psychotherapy for Adolescents (IPA)** One descriptive study explored Inpatient Psychotherapy for Adolescents (IPA) as a treatment for young people with Avoidant PD, Obsessive-Compulsive PD, Borderline PD, PD not otherwise specified, and any PD [68]. The intervention consisted of weekly individual and group sessions. A follow-up measure was implemented at baseline and 12-months. The intervention was successful at reducing PD symptoms at 12-month follow-up, where 28 from 65 participants reported change in scores.

The study did not report on presence or absence of adverse outcomes.

**Cognitive Emotion Regulation Strategies (CERS)** One pre-post study investigated weekly CERS as a treatment for young people with PDs [69]. No follow-up measures were included. CERS successfully decreased PD symptoms (*p* < 0.001).

The study did not report on presence or absence of adverse outcomes.

**Cutting Down Programme (CDP)** One prospective cohort group follow-up study examined ten sessions of CDP, a cognitive-behavioural short-term intervention aimed at reducing non-suicidal self-injuries, in comparison to TAU for adolescents with non-suicidal self-injuries with no follow-up measurement [70]. CDP was found to reduce adolescents meeting BPD criteria post-treatment (*p* < 0.001), but was not significantly different to TAU (*p* = 0.526). Additionally, no improvement in quality of life was found at post-treatment and follow-up (*p* = 0.639).

The study did not report on presence or absence of adverse outcomes.

**Online integrative trans-diagnostic treatment** A quasi-experimental pre-post study investigated eight online integrative trans-diagnostic treatment sessions as an intervention for emerging BPD with a 3-month follow up measurement [71]. The intervention was successful in reducing internet addiction and high-risk behaviours in participants with emerging BPD (*p* < 0.001; *p* < 0.001).

The study did not report on presence or absence of adverse outcomes.

**Road Maps** An acceptability and feasibility study examined a short-term group therapy (12 sessions with no follow-up measurement) for emerging BPD called Road Maps [72], delivered as a community/outpatient therapeutic group by the South Australia BPD Collaborative [73]. The intervention was found to be acceptable (drop-out rate of 27–38).

The study reported no deaths and a decrease in emergency department visits.

### Psychosocial preventions/Interventions

All studies focused on preventative interventions for emerging antisocial PD.

**Good Behaviour Game (GBG)** Two RCTs examined GBG as a preventive intervention for antisocial PD for students in the first and second grade with a follow-up measure at ages 19 to 21 years [74, 75]. GBG aims to create a social environment which teaches students to change their own and other student’s disruptive or aggressive behaviours (e.g. name-calling) in classrooms by dividing the class into teams. The team with the highest points wins and receives privileges such as extra free time [76]. GBG’s duration was not indicated, but the programme was held three times a week. GBG significantly reduced risk of highly aggressive disruptive presentations of ASPD in GBC group relative to controls (*p* = 0.02) [74]. The intervention was found to be predominantly significant in the persistent high class (cohort 1 males) (*p* < 0.001), in contrast to the persistent high and medium group in the cohort 1 females (*p* = 0.4959–0.7064) [75].

The studies did not report on presence or absence of adverse outcomes.

**Early Parent Training (The Incredible Years Programme)** One study explored if Incredible Years Programme training (average of 9 to 15 sessions) with parents would reduce antisocial PD in two populations: non-clinically referred children with severe problems (indicated) and children with milder problems (selective high-risk) [77]. The study included a long-term follow-up from four to 10 years. The programme demonstrated a reduction of ODD (which can precede antisocial PD) [78] in children within the indicated sample (*p* = 0.01). There were no significant benefits for children within the selective high-risk programme.

The study did not report on presence or absence of adverse outcomes.

### Pharmacological preventions/Interventions

Three RCTs examined pharmacological interventions for young people with BPD [79, 80] and a preventative treatment for emerging schizotypal PD [81].

**Omega-3 Fatty Acid** Amminger et al. (2013) [79] examined omega-3 (0.5 g) vs. a placebo pill containing coconut oil, over 12 weeks for young people with BPD and high risk for psychosis. Raine et al. (2020) [81] compared effects of CBT with omega-3, CBT alone, omega-3 alone, and TAU on 290 children meeting full or partial diagnosis for conduct or oppositional defiant disorder (ODD). Only Raine et al. (2024) [81] included a follow-up measure at three, six, and 12-months. Amminger et al. (2013) [79] found omega-3 acid successfully reduced BPD (*p* = 0.01), depression (*p* = 0.005), and anxiety (*p* = 0.03) symptoms across 12-weeks. Similarly, Raine et al. (2024) [81] found omega-3 resulted in a decrease in Schizotypal Personality Questionnaire-Child (SPQ-C) scores in comparison to controls at 3-month and 6-month follow-up respectively, with (3-months *p* = 0.013; 6-month *p* = 0.00003) or without CBT being present (3-months *p* = 0.0008; 6-months *p* = 0.003). Both studies investigated adverse events and found none [79] or minor [81] adverse effects following omega-3 supplementation. Raine et al. (2024) [81] had a small sample of participants (*n* = 18) who reported adverse effects including: diarrhoea, upset stomach, vomiting, nausea, feeling tired/sleepy, spots found on the face and headaches.

**Intranasal Oxytocin** One RCT [80] investigated intranasal oxytocin (24 IU total) vs. a placebo nasal spray on emerging BPD. Intranasal oxytocin was found to be an acceptable treatment (80–100% completion rate).

The study found no adverse events.

## Discussion

This review synthesised evidence from 43 studies on psychological, psychosocial and pharmacological treatments aimed at preventing or treating PDs in young people. BPD was the most frequently studied PD, and DBT emerged as the most commonly evaluated and acceptable intervention for its treatment. Across multiple studies, DBT demonstrated reductions in symptoms or criteria for BPD, with improvements observed both immediately post-treatment and at follow-up [41, 44–50]. Findings are consistent with previous systematic reviews that found DBT to be an effective treatment for adolescents [82] and adults with BPD [27, 83].

Depression and anxiety commonly co-occur with PDs and contribute to poor psychosocial outcomes [84, 85]. Depression and anxiety were frequently examined as secondary outcomes of intervention studies in which PD symptoms or traits were the primary outcomes. DBT was found to significantly reduce self-reported depression across several studies [44, 47, 51], which is consistent with adult BPD literature [83]. Findings related to anxiety were less encouraging; DBT did not significantly reduce anxiety post-treatment [46, 47, 51] despite evidence from other clinical populations suggesting DBT skills can help alleviate anxiety [86]. This discrepancy may indicate DBT does not adequately target anxiety-specific mechanisms in BPD in young people.

Four studies reported high completion rates for DBT among BPD [45, 47, 50, 51], although some observed moderate attrition [44]. Findings support the feasibility of delivering DBT as an intensive intervention, and challenge assumptions that adolescents with personality pathology cannot engage in structured therapy [87, 88].

Findings for MBT were more variable. Some studies reported MBT as more effective than TAU in reducing BPD and avoidant PD symptoms [53, 55], others found no significant benefit for BPD symptoms [54, 57]. The mixed findings contradict existing systematic reviews which find MBT to significantly reduce BPD symptoms [82]. One possible explanation is the limited number of MBT studies included in this review. Additionally, studies reporting poorer outcomes for BPD primarily evaluated MBT delivered exclusively in a group format. In terms of emotion regulation, MBT appeared to offer some benefit for avoidant PD, a condition often associated with pronounced social anxiety [43]. However, these findings were inconsistent, likely due to differences in treatment format, dosage, and study design.

Psychodynamic therapy and AIT showed promising results with large effect sizes for BPD symptom reduction and remission [41, 67]. These findings should be interpreted with caution due to limited number of high-quality studies available which underscores the need for further research beyond DBT and MBT. Generalisability of findings from this review are limited by variability in study quality and design.

Outcomes related to hospitalisation, emergency department visits, and inpatient service use were mixed. DBT and MBT found no significant advantage over TAU in reducing healthcare service use [46, 48, 57]. This contrasts with adult literature suggesting these therapies can reduce service dependence [89, 90]. Notably, DBT studies included participants with recent self-harm episodes, which may have led to extended aftercare and delayed discharge [91], inflating service use figures.

Evidence for process variables such as safety of novel interventions such as omega-3 supplementation [79, 81] and short-term specialised therapies [72] remains limited but promising. Future research should include more systematic monitoring of adverse effects and explore predictors of dropout, such as reflective functioning [24], to optimise retention and tailoring of interventions.

### Implications for future research

This review highlights a growing body of evidence supporting psychological interventions for young people with emerging personality difficulties, with DBT showing consistent benefits for acute clinical outcomes such as self-harm, suicidal ideation, and emotion dysregulation in young people with BPD. At the same time, much of the adolescent DBT literature, particularly studies emphasising early stabilisation and skills, was designed around these proximal outcomes. As a result, conclusions about change in broader domains (e.g., identity disturbance, interpersonal functioning, and attachment-related difficulties) are limited by outcome selection, follow-up length, and the specific phases/components delivered. Recent adaptations (e.g., DBT-Prolonged Exposure Protocol) introduce trauma-focused elements when indicated, underscoring that DBT can be integrated to address co-occurring post-traumatic presentations [92].

Approaches such as MBT, CAT, and psychodynamic therapies are theoretically oriented toward mentalising capacity [93], relational patterns [94], and self/identity process [95]; in some settings, these models are used to target longer-term change in interpersonal and personality functioning. Contemporary evidence indicates that personality disorders result from a blend of influences - genetic, neurobiological, attachment-related, and sometimes traumatic [96]. However, trauma is not seen as universally causal. Interventions such as MBT and CAT are grounded in developmental and attachment theory, which is particularly pertinent for individuals exhibiting early relational dysfunction. Taken together, the evidence suggests a pragmatic, staged or integrative strategy may be appropriate in certain cases, using DBT to reduce acute risk and enhance stabilisation, and considering additional or sequential interventions when the primary treatment goal shifts toward longer-term change in personality and relational functioning.

Current evidence base remains disproportionately focused on BPD in young people. Future research must extend its scope to examine effective treatments across the spectrum of personality pathology. Additionally, most studies recruited predominantly female participants, limiting generalisability of findings to male populations, which underscores the need for recruitment strategies that address potential barriers to engagement among underrepresented groups, such as stigma or late identification in males.

Much of the adolescent evidence base studies relies on small samples and non-randomised designs, with heterogeneous outcomes and limited follow-up. Well-powered, methodologically rigorous RCTs are needed to clarify effectiveness.

Many studies investigating psychological treatments did not examine and report on whether adverse outcomes were investigated [95]. Recommendation for future studies include examining and reporting on adverse events.

There remains a clear need for more research into psychosocial and pharmacological approaches as preliminary findings suggest potential benefits. However, limited number of studies and variability in sample sizes constrains the ability to draw firm conclusions. Future research should investigate a wider range of modalities, including family-based interventions, and culturally informed adaptations of existing treatments.

Future research should also consider the value of clinical staging frameworks, which emphasise early identification, subthreshold presentations, and matching intervention intensity to the individual’s stage of illness. In youth mental health, staging models have been used to guide stepped-care approaches that prioritise early, proportionate intervention and support timely escalation when symptoms or functional impairments progress [97]. Applying a staging perspective to emerging personality difficulties may help clarify when brief, skills-based approaches (such as DBT-skills or early-intervention variants) are sufficient, and when more comprehensive or relationally focused therapies (e.g., MBT or CAT) are indicated to address persistent interpersonal, identity, or attachment-related difficulties.

This review highlights the need for high-quality studies on prevention, especially given the limited number of rigorous studies found in the initial search. For example, a review [98] found early detection and screening may help reduce long-term negative outcomes of PDs. Scarcity of preventative studies may reflect the low proportion of young people (around 1%) who receive an PD diagnosis from mental health professionals [16]. Drozdova et al. (2023) [99] found over 60% of mental health clinicians treating young people found the term ‘emerging PD’ to be unhelpful to staff and families, which may add obstacles to preventative approaches being studied.

### Strengths and limitations

This review has notable strengths. Consultation with a clinical psychologist and a service user with lived experience ensured relevance to real-world practice and alignment with service-user priorities. The review adopted a rigorous methodological approach by focusing on moderate-to-high quality studies, which allowed for a more reliable synthesis of findings across diverse study designs. Importantly, the review included studies across a range of therapeutic modalities and diagnostic labels rather than restricting inclusion to one specific PD, broadening its clinical applicability.

Several limitations of this review should be acknowledged. We did not conduct a meta-analysis; given the small number of new RCTs since the Jørgensen et al. meta-analysis (2021) [24] and the considerable diversity in outcomes, it would likely be underpowered and risk producing misleading conclusions. Our inclusion of search terms such as DBT and MBT may have introduced bias toward BPD and these therapeutic modalities, which dominated the sample. Limiting the search to English may have introduced language bias as only two studies were located in non-native English-speaking countries. The EPHPP tool threshold is relatively strict and combined with our protocol decision to exclude low-quality studies (*n* = 42), it likely contributed to a higher proportion of moderate/high ratings among studies. In contrast, traditional risk-of-bias tools, such as those used in Jørgensen et al., (2021) [24] often rate studies as high risk if any critical domain fails, resulting in lower overall quality ratings. These methodological differences should be considered when interpreting findings, as moderate quality still indicates some limitations.

Finally, 161 full-text papers were excluded due to lack of author response for age-related data, which may have further narrowed the scope of included findings.

## Conclusion

Overall, this review provides evidence that early intervention can reduce symptoms and associated impairments in PDs in young people. However, the field remains limited by a narrow diagnostic and demographic focus towards BPD in young people. Future research should expand the scope of interventions, populations, and outcome domains to ensure more inclusive, equitable, and effective treatment pathways for youth experiencing early signs of personality pathology.

## Supplementary information

Below is the link to the electronic supplementary material.

Supplementary File 1 (DOCX 267 KB)

## Data Availability

No datasets were generated or analysed during the current study.

## Declarations

### Trial registration, prospero registration number

CRD42020202035

## References

[1] Clark LA, Jan (2007) Assessment and diagnosis of personality disorder: perennial issues and an emerging reconceptualization. Annu Rev Psychol 58(1):227–257. 10.1146/annurev.psych.57.102904.190200 16903806

[2] Videler AC, Hutsebaut J, Schulkens JEM, Sobczak S, Van Alphen SPJ (2019) A life span perspective on borderline personality disorder. Curr Psychiatry Rep 21(7). 10.1007/s11920-019-1040-1 PMC6546651 31161404

[3] De Clercq B, De Fruyt F, Widiger TA (2009) Integrating a developmental perspective in dimensional models of personality disorders. Clin Psychol Rev 29(2):154–162. 10.1016/j.cpr.2008.12.002 19167138

[4] Kongerslev MT, Chanen AM, Simonsen E (2014) Personality disorder in childhood and adolescence comes of age: A review of the current evidence and prospects for future research. Scand J Child Adolesc Psychiatry Psychol 3(1):31–48. 10.21307/sjcapp-2015-004

[5] Johnson JG, Bromley E, Bornstein RF, Sneed JR (2006) Personality Disorders. In: Wolfe DA, Mash EJ (eds) Behavioral and emotional disorders in children and adolescents: Nature, assessment, and treatment. Guilford, Eds, New York, pp 463–484

[6] Fontana A, De Panfilis C, Casini E, Preti E, Richetin J, Ammaniti M (2018) Rejection sensitivity and psychopathology symptoms in early adolescence: The moderating role of personality organization. J Adolesc 67(1):45–54. 10.1016/j.adolescence.2018.05.012 29906692

[7] Chanen AM, McCutcheon LK, Jovev M, Jackson HJ, McGorry PD (2007) Prevention and early intervention for borderline personality disorder. Med J Aust 187. 10.5694/j.1326-5377.2007.tb01330.x 17908019

[8] Dalsgaard S et al (2020) Association of mental disorder in childhood and adolescence with subsequent educational achievement. JAMA Psychiatry 77(8):797. 10.1001/jamapsychiatry.2020.0217 PMC7097843 32211833

[9] Ciocca G et al (2023) Sexual dysfunctions and problematic sexuality in personality disorders and pathological personality traits: A systematic review. Curr Psychiatry Rep 25(3):93–103. 10.1007/s11920-023-01409-9 PMC9968244 36738436

[10] Moran P et al (2016) The influence of personality disorder on the future mental health and social adjustment of young adults: a population-based, longitudinal cohort study. Lancet Psychiatry 3(7):636–645. 10.1016/s2215-0366(16)30029-3 27342692

[11] Livanou M, Furtado V, Winsper C, Silvester A, Singh SP (2019) Prevalence of mental disorders and symptoms among incarcerated youth: A meta-analysis of 30 studies. Int J Forensic Ment Health 18(4):400–414. 10.1080/14999013.2019.1619636

[12] Emerging Personality Disorder (2017) In child psychology and psychiatry, 1st edn. Wiley, pp 255–262. 10.1002/9781119170235.ch29

[13] Ayodeji E et al (2015) The influence of personality disorder on outcome in adolescent self-harm. Br J Psychiatry 207(4):313–319. 10.1192/bjp.bp.113.138941 26250743

[14] Laurenssen EMP, Hutsebaut J, Feenstra DJ, Van Busschbach JJ, Luyten P (2013) Diagnosis of personality disorders in adolescents: A study among psychologists. Child Adolesc Psychiatry Ment Health 7(1). 10.1186/1753-2000-7-3 PMC3583803 23398887

[15] Kingsley D (2022) Debate: Child and adolescent mental health professionals have a responsibility to diagnose personality disorder. Child Adolesc Ment Health 27(2):196–198. 10.1111/camh.12557 35365896

[16] Chanen AM, Nicol K (2021) Five failures and five challenges for prevention and early intervention for personality disorder. Curr Opin Psychol 37:134–138. 10.1016/j.copsyc.2020.12.005 33513519

[17] Marriner L, Larkin M, Urquhart Law G, Kaur Bhogal S (2025) I’m ruined: Young people’s and their mothers’ experiences of living with, and receiving a diagnosis of, borderline personality disorder: An interpretative phenomenological analysis. Psychol Psychother Theory Res Pract 98(2):361–372. 10.1111/papt.12549 PMC12065063 39373270

[18] Chanen AM (2022) Debate: It is time to stop turning a blind eye to personality disorder in young people. Child Adolesc Ment Health 27(2):194–195. 10.1111/camh.12554 35293112

[19] Fossati A, Somma A (2021) The assessment of personality pathology in adolescence from the perspective of the alternative DSM-5 model for personality disorder. Curr Opin Psychol 37:39–43. 10.1016/j.copsyc.2020.07.015 32827876

[20] Vickers G, Combes H, Lonsdale J (2023) Mental health professionals’ views of the borderline personality disorder diagnosis for children and young people: a Q-methodological study. J Ment Health Train Educ Pract 18(1):1–13. 10.1108/jmhtep-09-2021-0104

[21] Hutsebaut J, Clarke SL, Chanen AM (May 2023) The diagnosis that should speak its name: why it is ethically right to diagnose and treat personality disorder during adolescence. Front Psychiatry 14:1130417. 10.3389/fpsyt.2023.1130417 PMC10203159 37229381

[22] Palermo L, Cavelti M, Sele S, Sharp C, Reichl C, Kaess M (2025) Personality functioning improvements in adolescents from an early intervention clinic for personality disorders. Eur Child Adolesc Psychiatry. 10.1007/s00787-025-02913-4 PMC13219104 41212286

[23] Lee NK, Cameron J, Jenner L (2015) A systematic review of interventions for co-occurring substance use and borderline personality disorders. Drug Alcohol Rev 34(6):663–672. 10.1111/dar.12267 25919396

[24] Jørgensen MS, Storebø OJ, Stoffers-Winterling JM, Faltinsen E, Todorovac A, Simonsen E (2021) Psychological therapies for adolescents with borderline personality disorder (BPD) or BPD features—A systematic review of randomized clinical trials with meta-analysis and Trial Sequential Analysis. PLoS ONE 16(1):e0245331. 10.1371/journal.pone.0245331 PMC7808579 33444397

[25] Bourvis N, Cohen D, Benarous X (2023) Therapeutic and preventive interventions in adolescents with borderline personality disorder: Recent findings, current challenges, and future directions. J Clin Med 12(20):6668. 10.3390/jcm12206668 PMC10607502 37892806

[26] Wong J, Bahji A, Khalid-Khan S (2020) Psychotherapies for adolescents with subclinical and borderline personality disorder: A systematic review and meta-analysis. Can J Psychiatry 65(1):5–15. 10.1177/0706743719878975 PMC6966252 31558033

[27] Stoffers-Winterling JM et al (2022) Psychotherapies for borderline personality disorder: a focused systematic review and meta-analysis. Br J Psychiatry 221(3):538–552. 10.1192/bjp.2021.204 35088687

[28] Sharp C, Rossouw T (2024) Mentalization-based treatment for adolescents (MBT-A). Psychodyn Psychiatry 52(4):542–562. 10.1521/pdps.2024.52.4.542 39679703

[29] Simonsen S, Bo S, Sharp C (2026) Mentalization-based therapy for personality disorder in adolescents. Curr Opin Psychiatry 39(1):67–71. 10.1097/YCO.0000000000001033 40709661

[30] De Andrade D et al (2024) Randomized effectiveness-implementation trial of dialectical behavior therapy interventions for young people with borderline personality disorder symptoms. J Clin Psychol 80(10):2117–2133. 10.1002/jclp.23725 38874116

[31] Kwan B, Rickwood DJ (2015) A systematic review of mental health outcome measures for young people aged 12 to 25 years. BMC Psychiatry 15(1). 10.1186/s12888-015-0664-x PMC4647516 26573269

[32] Sawyer SM, Azzopardi PS, Wickremarathne D, Patton GC (2018) The age of adolescence. Lancet Child Adolesc Health 2(3):223–228. 10.1016/s2352-4642(18)30022-1 30169257

[33] Singh V, Kumar A, Gupta S (2022) Mental health prevention and promotion—a narrative review. Front Psychiatry 13:898009. 10.3389/fpsyt.2022.898009 PMC9360426 35958637

[34] Costello EJ (2016) Early detection and prevention of mental health problems: developmental epidemiology and systems of support. J Clin Child Adolesc Psychol 45(6):710–717. 10.1080/15374416.2016.1236728 27858462

[35] APA Dictionary of Psychology, Intervention. [Online]. Available: https://dictionary.apa.org/intervention

[36] Covidence Veritas Health Innovation (2024). [Online]. Available: https://www.covidence.org

[37] Armijo‐Olivo S, Stiles CR, Hagen NA, Biondo PD, Cummings GG (2012) Assessment of study quality for systematic reviews: A comparison of the cochrane collaboration risk of bias tool and the effective public health practice project quality assessment tool: Methodological research. J Eval Clin Pract 18(1):12–18. 10.1111/j.1365-2753.2010.01516.x 20698919

[38] Nadelson S, Nadelson LS (2014) Evidence-based practice article reviews using CASP tools: A method for teaching EBP. Worldviews evid. Based Nurs 11(5):344–346. 10.1111/wvn.12059 25156640

[39] Popay J et al (2006) Guidance on the conduct of narrative synthesis in systematic reviews

[40] Andersen CF, Poulsen S, Fog-Petersen C, Jørgensen MS, Simonsen E (2021) Dropout from mentalization-based group treatment for adolescents with borderline personality features: A qualitative study. Psychother Res 31(5):619–631. 10.1080/10503307.2020.1813914 32878583

[41] Schmeck K et al (2023) Effectiveness of adolescent identity treatment (AIT) versus DBT-a for the treatment of adolescent borderline personality disorder. Personal Disord Theory Res Treat 14(2):148–160. 10.1037/per0000572 35587408

[42] Lakeman R, Emeleus M (2020) The process of recovery and change in a dialectical behaviour therapy programme for youth. Int J Ment Health Nurs 29(6):1092–1100. 10.1111/inm.12749 32535985

[43] Lind M, Kiel L, Hansen SB, Jørgensen MS, Simonsen E (2023) Narrative identity within mentalization-based group therapy for adolescents: A feasibility study. Children. 10(5):854. 10.3390/children10050854 PMC10217683 37238402

[44] Pistorello J, Fruzzetti AE, MacLane C, Gallop R, Iverson KM (2012) Dialectical behavior therapy (DBT) applied to college students: A randomized clinical trial. J Consult Clin Psychol 80(6):982–994. 10.1037/a0029096 PMC3514574 22730955

[45] Lyng J, Swales MA, Hastings RP, Millar T, Duffy DJ (2020) Outcomes for 18 to 25-year‐olds with borderline personality disorder in a dedicated young adult only DBT programme compared to a general adult DBT programme for all ages 18+. Early Interv Psychiatry 14(1):61–68. 10.1111/eip.12808 31044536

[46] Phillips MD, Parham R, Hunt K, Camp J (2024) Dialectical behaviour therapy outcomes for adolescents with autism spectrum conditions compared to those without: findings from a seven-year service evaluation. Adv Autism 10(3):185–199. 10.1108/aia-05-2023-0021

[47] Moran LR et al (2018) Treatment effects following residential dialectical behavior therapy for adolescents with borderline personality disorder. Evid.-Based Pract Child Adolesc Ment Health 3(2):117–128. 10.1080/23794925.2018.1476075 PMC6377246 30778398

[48] Mehlum L et al (2016) Dialectical behavior therapy compared with enhanced usual care for adolescents with repeated suicidal and self-harming behavior: outcomes over a one-year follow-up. J Am Acad Child Adolesc Psychiatry 55(4):295–300. 10.1016/j.jaac.2016.01.005 27015720

[49] Courtney DB, Flament MF (2015) Adapted dialectical behavior therapy for adolescents with self-injurious thoughts and behaviors. J Nerv Ment Dis 203(7):537–544. 10.1097/nmd.0000000000000324 26075841

[50] Buerger A, Fischer-Waldschmidt G, Hammerle F, Auer KV, Parzer P, Kaess M (2019) Differential change of borderline personality disorder traits during dialectical behavior therapy for adolescents. J Personal Disord 33(1):119–134. 10.1521/pedi_2018_32_334 30036173

[51] Denaro J (2008) Effectiveness of dialectical behavior therapy in the treatment of adolescent girls with borderline personality features in an outpatient community mental health setting. Diss Abstr Int Sect B Sci Eng

[52] Schiffler T, Seiler-Ramadas R, Štefanac S, Haider S, Mües HM, Grabovac I (2022) Implementation of a mobile DBT app and its impact on suicidality in transitional age youth with borderline personality disorder: A qualitative study. Int J Environ Res Public Health 19(2):701. 10.3390/ijerph19020701 PMC8775851 35055523

[53] Rossouw TI, Fonagy P (2012) Mentalization-based treatment for self-harm in adolescents: A randomized controlled trial. J Am Acad Child Adolesc Psychiatry 51(12):1304–1313. 10.1016/j.jaac.2012.09.018 23200287

[54] Simonsen E, Vestergaard M, Storeb⊘ OJ, Bo S, J⊘rgensen MS (2021) Prediction of treatment outcome of adolescents with borderline personality disorder: A 2-year follow-up study. J Personal Disord 3(Supplement B):111–130. 10.1521/pedi_2021_35_524 33999658

[55] Bo S, Bateman A, Kongerslev MT (2019) Mentalization-based group therapy for adolescents with avoidant personality disorder: adaptations and findings from a practice-based pilot evaluation. J Infant Child Adolesc Psychother 18(3):249–262. 10.1080/15289168.2019.1625655

[56] Hauber K, Boon AE, Vermeiren R (2017) Examining changes in personality disorder and symptomology in an adolescent sample receiving intensive mentalization based treatment: A pilot study. Child Adolesc Psychiatry Ment Health 11(1). 10.1186/s13034-017-0197-9 PMC5706392 29209413

[57] Beck E et al (2020) Mentalization-based treatment in groups for adolescents with borderline personality disorder: A randomized controlled trial. J Child Psychol Psychiatry 61(5):594–604. 10.1111/jcpp.13152 31702058

[58] Popolo R et al (2018) Metacognitive interpersonal therapy in group: A feasibility study. Res Psychother Psychopathol Process Outcome 21(3). 10.4081/ripppo.2018.338 PMC7451332 32913773

[59] Popolo R et al (2019) Metacognitive Interpersonal Therapy in group (MIT-G) for young adults with personality disorders: A pilot randomized controlled trial. Psychol Psychother Theory Res Pract 92(3):342–358. 10.1111/papt.12182 29624832

[60] Popolo R et al (2022) Metacognitive interpersonal therapy in group versus TAU + waiting list for young adults with personality disorders: Randomized clinical trial. Personal Disord Theory Res Treat 13(6):619–628. 10.1037/per0000497 34383540

[61] Chanen AM et al (2008) Early intervention for adolescents with borderline personality disorder using cognitive analytic therapy: randomised controlled trial. Br J Psychiatry 193(6):477–484. 10.1192/bjp.bp.107.048934 19043151

[62] Chanen AM et al (2009) Early Intervention for Adolescents with Borderline Personality Disorder: Quasi-Experimental Comparison with Treatment as Usual. Aust N Z J Psychiatry 43(5):397–408. 10.1080/00048670902817711 19373700

[63] Chanen AM et al (2022) Effect of 3 forms of early intervention for young people with borderline personality disorder: The MOBY randomized clinical trial. JAMA Psychiatry 79(2):109. 10.1001/jamapsychiatry.2021.3637 PMC8674805 34910093

[64] Gleeson JFM, Chanen A, Cotton SM, Pearce T, Newman B, McCutcheon L (2012) Treating co-occurring first‐episode psychosis and borderline personality: a pilot randomized controlled trial. Early Interv Psychiatry 6(1):21–29. 10.1111/j.1751-7893.2011.00306.x 22379625

[65] Schuppert HM et al (2009) Effectiveness of an emotion regulation group training for adolescents—a randomized controlled pilot study. Clin Psychol Psychother 16(6):467–478. 10.1002/cpp.637 19630069

[66] Marieke Schuppert H et al (2012) Emotion regulation training for adolescents with borderline personality disorder traits: A Randomized controlled trial. J Am Acad Child Adolesc Psychiatry 51(12):1314–1323. 10.1016/j.jaac.2012.09.002 23200288

[67] Salzer S, Cropp C, Streeck-Fischer A (2014) Early Intervention for borderline personality disorder: Psychodynamic therapy in adolescents. Z Für Psychosom Med Psychother 60(4):368–382. 10.13109/zptm.2014.60.4.368 25528872

[68] Feenstra DJ, Laurenssen EMP, Timman R, Verheul R, Busschbach JJV, Hutsebaut J (2014) Long-term outcome of inpatient psychotherapy for adolescents (IPA) with personality pathology. J Personal Disord 28(5):637–656. 10.1521/pedi_2014_28_132 24689768

[69] Boon AE, Hauber K (2022) Cognitive emotion regulation strategies in relation to treatment outcome in a clinical sample of adolescents with personality disorders. Clin Psychol Psychother 29(6):1897–1904. 10.1002/cpp.2758 PMC10084372 35653420

[70] Rockstroh F et al (2023) Brief Psychotherapeutic Intervention Compared with Treatment as Usual for Adolescents with Nonsuicidal Self-Injury: Outcomes over a 2–4-Year Follow-Up. Psychother Psychosom 92(4):243–254. 10.1159/000531092 PMC10568598 37487473

[71] Mohamadpour F, Mohammadi N (2024) Effectiveness of online integrative trans-diagnostic treatment on internet addiction and high-risk behaviors in female adolescents with borderline personality disorder with comorbid depressive disorder. Front Psychiatry 14. 10.3389/fpsyt.2023.1291579 PMC10797106 38250259

[72] Bartsch DR, Cooke-O’Connor L, Connerty TJ, Van Roekel H, Cammell P (2024) Acceptability and feasibility of a short-term group therapy for people with borderline personality disorder symptoms. Australas Psychiatry 32(4):330–335. 10.1177/10398562241246485 PMC11318202 38621694

[73] Borderline personality disorder collaborative programs [Online]. Available: https://www.sahealth.sa.gov.au/wps/wcm/connect/public+content/sa+health+internet/services/mental+health+and+drug+and+alcohol+services/mental+health+services/statewide+specialist+services/borderline+personality+disorder+collaborative/borderline+personality+disorder+services/borderline+personality+disorder+services

[74] Kellam SG et al (2008) Effects of a universal classroom behavior management program in first and second grades on young adult behavioral, psychiatric, and social outcomes. Drug Alcohol Depend 95:S5–S28. 10.1016/j.drugalcdep.2008.01.004 PMC2512256 18343607

[75] Petras H, Kellam SG, Brown CH, Muthén BO, Ialongo NS, Poduska JM (2008) Developmental epidemiological courses leading to antisocial personality disorder and violent and criminal behavior: Effects by young adulthood of a universal preventive intervention in first- and second-grade classrooms. Drug Alcohol Depend 95:S45–S59. 10.1016/j.drugalcdep.2007.10.015 PMC2706504 18243581

[76] Tingstrom DH, Sterling-Turner HE, Wilczynski SM (2006) The good behavior game: 1969–2002. Behav Modif 30(2):225–253. 10.1177/0145445503261165 16464846

[77] Scott S, Briskman J, O’Connor TG (2014) Early prevention of antisocial personality: Long-term follow-up of two randomized controlled trials comparing indicated and selective approaches. Am J Psychiatry 171(6):649–657. 10.1176/appi.ajp.2014.13050697 24626738

[78] Ghosh A, Ray A, Basu A (2017) Oppositional defiant disorder: current insight. Psychol Res Behav Manag 10:353–367. 10.2147/prbm.s120582 PMC5716335 29238235

[79] Amminger GP et al (2013) Omega-3 fatty acid supplementation in adolescents with borderline personality disorder and ultra-high risk criteria for psychosis: A post hoc subgroup analysis of a double—blind, randomized controlled trial. Can J Psychiatry 58(7):402–408. 10.1177/070674371305800705 23870722

[80] Ha C (201 6) The effects of intranasal oxytocin on social cognitive functioning in adolescents with borderline personality disorder compared to a sample of non-clinical adolescents. Diss Abstr Int Sect B Sci Eng

[81] Raine A, Gur RC, Gur RE, Richmond TS, Hibbeln J, Liu J (2024) Omega-3 Supplementation reduces schizotypal personality in children: A randomized controlled trial. Schizophr Bull 50(5):1117–1126. 10.1093/schbul/sbae009 PMC11349019 38300759

[82] Johnstone OK, Marshall JJ, McIntosh LG (2022) A review comparing dialectical behavior therapy and mentalization for adolescents with borderline personality traits, suicide and self-harming behavior. Adolesc Res Rev 7(2):187–209. 10.1007/s40894-020-00147-w

[83] Cristea IA, Gentili C, Cotet CD, Palomba D, Barbui C, Cuijpers P (2017) Efficacy of psychotherapies for borderline personality disorder: A Systematic review and meta-analysis. JAMA Psychiatry 74(4):319. 10.1001/jamapsychiatry.2016.4287 28249086

[84] Friborg O, Martinsen EW, Martinussen M, Kaiser S, Øvergård KT, Rosenvinge JH (2014) Comorbidity of personality disorders in mood disorders: A meta-analytic review of 122 studies from 1988 to 2010. J Affect Disord 152–154. 10.1016/j.jad.2013.08.023 24120406

[85] Newton-Howes G et al (2014) Influence of personality on the outcome of treatment in depression: Systematic review and meta-analysis. J Personal Disord 28(4):577–593. 10.1521/pedi_2013_27_070 24256103

[86] Webb CA, Beard C, Kertz SJ, Hsu KJ, Björgvinsson T (2016) Differential role of CBT skills, DBT skills and psychological flexibility in predicting depressive versus anxiety symptom improvement. Behav Res Ther 81:12–20. 10.1016/j.brat.2016.03.006 PMC4860036 27057997

[87] De Freixo Ferreira L, Guerra C, Vieira-Coelho MA (2023) Predictors of psychotherapy dropout in patients with borderline personality disorder: A systematic review. Clin Psychol Psychother 30(6):1324–1337. 10.1002/cpp.2888 37522280

[88] Arntz A et al (2023) Dropout from psychological treatment for borderline personality disorder: a multilevel survival meta-analysis. Psychol Med 53(3):668–686. 10.1017/s0033291722003634 PMC9975988 36453183

[89] Coyle TN, Shaver JA, Linehan MM (2018) On the potential for iatrogenic effects of psychiatric crisis services: The example of dialectical behavior therapy for adult women with borderline personality disorder. J Consult Clin Psychol 86(2):116–124. 10.1037/ccp0000275 29369662

[90] Bales D et al (2012) Treatment outcome of 18-month, day hospital mentalization-based treatment (MBT) in patients with severe borderline personality disorder in the Netherlands. J Personal Disord 26(4):568–582. 10.1521/pedi.2012.26.4.568 22867507

[91] Diggins E, Kelley R, Cottrell D, House A, Owens D (2017) Age-related differences in self-harm presentations and subsequent management of adolescents and young adults at the emergency department. J Affect Disord 208:399–405. 10.1016/j.jad.2016.10.014 27810724

[92] Harned MS, Gallop RJ, Valenstein-Mah HR (2018) What changes when? The course of improvement during a stage-based treatment for suicidal and self-injuring women with borderline personality disorder and PTSD. Psychother Res 28(5):761–775. 10.1080/10503307.2016.1252865 PMC6013413 27808001

[93] Clarke S, Thomas P, James K (2013) Cognitive analytic therapy for personality disorder: randomised controlled trial. Br J Psychiatry 202(2):129–134. 10.1192/bjp.bp.112.108670 23222038

[94] Clarkin JF, Meehan KB, De Panfilis C, Doering S (2023) Empirical developments in transference-focused psychotherapy. Am J Psychother 76(1):39–45. 10.1176/appi.psychotherapy.20220017 36221977

[95] Duggan C, Parry G, McMurran M, Davidson K, Dennis J (2014) The recording of adverse events from psychological treatments in clinical trials: Evidence from a review of NIHR-funded trials. Trials 15(1):335. 10.1186/1745-6215-15-335 PMC4152561 25158932

[96] Mei C, McGorry PD, Hickie IB (2019) Clinical Staging and Its Potential to Enhance Mental Health Care. In: McGorry PD, Hickie IB (eds) Clinical Staging in Psychiatry: Making Diagnosis Work for Research and Treatment. Cambridge University Press, pp 12–33

[97] Hickie IB, Carpenter JS, Iorfino F, Scott E, Cross S, Hermens DF (2019) The Utility of Clinical Staging in Youth Mental Health Settings: Neurobiological and Longitudinal Data from Sydney-Based Studies of Transdiagnostic Cohorts. In: McGorry PD, Hickie IB (eds) Clinical Staging in Psychiatry, 1st edn. Cambridge University Press, Eds, pp 81–102. doi: 10.1017/9781139839518.005.

[98] Bozzatello P, Garbarini C, Rocca P, Bellino S (2021) Borderline personality disorder: risk factors and early detection. Diagnostics 11(11):2142. 10.3390/diagnostics11112142 PMC8620075 34829488

[99] Drozdova Z, Bentham C, Collin T, Youakim S (2023) How helpful is the label of emerging personality disorder? A service evaluation exploring CAMHS clinicians’ experiences of young people being labelled with a diagnosis of emerging personality disorder. BJPsych Open 9:S133–S133. 10.1192/bjo.2023.369

